# Adverse Life Events and Depressive Symptoms in African American Youth: The Role of Control-Related Beliefs

**DOI:** 10.1155/2011/871843

**Published:** 2010-12-15

**Authors:** Yadira M. Sanchez, Sharon F. Lambert, Nicholas S. Ialongo

**Affiliations:** ^1^Department of Psychology, George Washington University, 2125 G Street NW, Washington, DC 20052, USA; ^2^Department of Mental Health, Johns Hopkins Bloomberg School of Public Health, Baltimore, MD 21205, USA

## Abstract

The association between experiences of adverse life events and adolescent depressive symptoms has been well documented. However, this association is not consistently observed in urban and low income African American youth. In addition, mechanisms linking life event stress and African American adolescents' depressive symptoms have received little attention. This study examined past year violent and nonviolent life events assessed in 6th grade as predictors of 7th grade depressive symptoms among a community epidemiologically defined sample of 447 (47% girls) urban African American adolescents. Depressive symptoms were assessed twice, at a 1-year interval, and initial depressive symptoms were controlled in the analyses. Control-related beliefs were examined as mediators of the association between life events and depressive symptoms, and gender was examined as a moderator of the association between control-related beliefs and depressive symptoms. Associations among study variables were examined in a series of models, from general to more specific. A model in which nonviolent and violent life events were examined separately and control and contingency beliefs examined as one latent variable was the most informative about the etiology of depressive symptoms in a sample of urban, African American youth. Implications of the findings for preventive interventions and future research are discussed.

## 1. Adverse Life Events and Depressive Symptoms in African American Youth: The Role of Control-Related Beliefs

Adolescent depression is a serious mental health issue that has been linked with several serious consequences such as academic difficulties, interpersonal difficulties, substance use problems, and the development of comorbid symptoms and disorders including anxiety and disruptive behavior disorders [1]. Thus, there has been interest in understanding the factors associated with adolescent depression in order to inform prevention and treatment efforts. Etiological research has identified adverse life events as important factors in the development of adolescent depression (e.g., [2–5]). While this link between stress and depression has been well documented, life event stress has not consistently been linked with depressive symptoms for urban and low income youth despite their high rates of exposure to adverse life events [4, 6]. Moreover, it is not clear whether similar mechanisms influence the development and maintenance of depression in response to experiences of adverse life events for these youth as compared to youth from other backgrounds [4]. This study examines control-related beliefs as a potential mechanism through which experiences of adverse life events may lead to the development of depressive symptoms in urban African American youth. Results from this research will help clarify questions about the etiology and maintenance of depressive symptoms in these youth and inform the design of culturally relevant interventions for African American youth.

### 1.1. Life Event Stress and Depressive Symptoms in Adolescents

Numerous studies have found that life event stress predicts increases in depressive symptoms, syndromes, and disorders in adolescents (e.g., [2–5, 7]). In fact, life event stress has been shown to be a stronger predictor of depressive syndromes and disorders than depressed mood [7]. Patton et al. [8] found that adolescents who experienced one negative life event were at a fivefold increased risk of developing stable Major Depressive Disorder compared to controls. Those who experienced multiple negative events had an eightfold increase in risk of developing stable depressive disorder [8]. One process by which negative life events can lead to increases in youth depressive outcomes is that major life events can lead to other, more enduring, daily stressors. 

Some of the available research with low-income, minority youth supports the link between adverse life events and depression (e.g., [4, 9]). For example, there is evidence that negative life events across a variety of domains, including economic, family, peer, discrimination, neighborhood/violence, and school domains, are positively associated with depressive symptoms in ethnically diverse urban youth in cross-sectional and prospective studies [4, 10]. On the other hand, other research suggests that urban African American youth residing in dangerous contexts are less likely to display their distress through internalizing behaviors than externalizing behaviors [11]; for example, they may express distress in ways that are less likely to be viewed as a sign of weakness (e.g., crying) [12]. Relatedly, the type of life events youth experience can impact whether or not they display depressive symptoms in response to adverse life event stress [11, 13]. For example, research suggests that violent life events are related to youth externalizing symptoms, while nonviolent life events are related to youth internalizing symptoms [14]. These differences in youth adjustment based on the type of life event experienced suggest that it is important to consider the type of life event experienced when examining the link between life events and depressive symptoms, and the distinction between violent and nonviolent life events appears to be important for understanding specificity in responses to stress.

### 1.2. Cognitive Theories Explaining Link between Life Event Stress and Depressive Symptoms

Cognitive theories of depression highlight the controllability of stressors to explain the stress-depression link. For example, learned helplessness theory [15] posits that individuals who experience life events perceived to be beyond their control may develop an expectation that future life events also will be uncontrollable, leading to a series of cognitive and motivational deficits resulting in depressed affect [15]. Similarly, the hopelessness theory of depression [16] proposes that some people characteristically infer that negative life events are caused by internal, stable, and global forces. Beck's cognitive theory of depression posits that some individuals have a negative cognitive schema, characterized by a negative view of themselves, the world, and the future, which is characteristic of depression and can be triggered by individuals' experiences of adverse life events [17]. The contingency-competence-control (C-C-C) model integrates these types of cognitions into one model [18, 19]. This model posits that perceived control, the perception that one has the ability to produce desired outcomes, is influenced by both contingency, perceptions about the degree to which an outcome is dependent on the behavior of people in general, and competence, perception of one's ability to produce the desired outcome [18, 20]. While perceived control and perceived competence are perceptions about the self, perceived contingency involves perceptions about youth in general (e.g., other African American adolescents). According to the C-C-C model, perceived contingency and competence are expected to significantly predict, but not fully account for, perceived control, since other factors also may contribute to perceived control [18]. 

Studies based on the C-C-C model have produced mixed results about the role that perceived contingency and control play in depression development. In a cross-sectional study of ethnically diverse adolescents ages 8 to 17, Weisz et al. [18] found that perceived contingency and perceived competence were significantly associated with perceived control. Furthermore, perceived contingency and competence were associated with adolescent depression, but perceived control was not. In their prospective study of predominantly Caucasian adolescents ages 10–14, Muris et al. [20] found that perceived contingency and perceived competence predicted perceived control. However, unlike Weisz et al. [18] study, perceived competence and perceived control predicted depression, but perceived contingency did not. These studies show that perceived control is influenced by perceived contingency and perceived competence. However, due to the mixed findings regarding the direct relationship of perceived control and contingency on depression in these studies, it is unclear what common and specific roles control and contingency beliefs may play in adolescent depressive symptoms. For this reason, Han et al. [21] have noted that it is important to examine the specific relations of different types of control-related beliefs (e.g., control and contingency) with internalizing symptoms. Thus, the present study examines general and specific associations between control and contingency beliefs and depressive symptoms to better understand the role of these cognitions in urban, African American youth. 

The few studies examining the applicability of cognitive theories of depression to low-SES, ethnically diverse youth suggest that control cognitions do affect depression development in these youth. Reinemann and Teeter Ellison [22] found that when youth experienced low levels of negative life events, those with a more internal locus of control reported lower levels of anhedonia than those with an external locus of control. However, for youth who experienced high levels of negative life events, an internal locus of control did not have these buffering effects; specifically, youth with elevated negative life events reported similar rates of anhedonia whether or not they had an internal locus of control. Thus, under conditions of high stress, an internal locus of control may be less able to buffer against depressive symptoms. In contrast, Cowen et al. [23] found that urban youth who displayed resilience in the face of highly stressful conditions reported significantly more use of internal locus of control than nonresilient youth. These studies suggest that an internal locus of control may be an important resource for youth exposed to adverse life events, but these protective effects may be limited for urban, minority youth living under highly stressful conditions. 

In addition to mixed findings regarding the role of cognitions in depression development in minority youth, methodological problems limit our understanding of how control cognitions affect psychological adjustment in these youth. Because studies have used different terminology for conceptually similar cognitions (e.g., internal and external locus of control; contingency, competence, and control beliefs), it is difficult to determine which types of control cognitions predict the development of youth depressive symptoms. While exceptions exist (e.g., [4, 22]), most studies have examined control-related cognitions with predominately Caucasian and middle class samples; thus, it is difficult to determine whether and how control-related beliefs play a role in the development of depression in low SES, ethnically diverse samples. For example, ethnic minority youth living in disadvantaged neighborhoods may perceive that others similar to themselves have little control over their environment given their knowledge of many uncontrollable events experienced by similar others (e.g., victimization by violence). If so, contingency beliefs may be particularly relevant for these youth. Finally, studies examining the role of control-related beliefs in depression development primarily have used cross-sectional designs, limiting our knowledge about whether these cognitions lead to changes in depressive symptoms.

### 1.3. Present Study

It is recognized that adverse life events can lead to low perceptions of control and contingency. This may be particularly true for youth living in disadvantaged neighborhoods, who often experience life events beyond their control (e.g., neighborhood violence) and may come to believe that their behaviors do not necessarily yield desired life outcomes; these lowered control beliefs may be linked to depressive symptoms. Thus, the goal of the current study was to examine whether control-related beliefs mediate the relationship between adverse life events and depressive symptoms in a sample of urban, low-SES, African American adolescents. Because prior research has found that nonviolent life events are more strongly associated with youth internalizing symptoms than violent life events [14], nonviolent and violent life events were examined separately. Prior research has shown that gender differences in cognitive styles, such as increased use of rumination in girls, help to explain the gender difference in adolescent depression [24–26]. Similarly, gender differences in control-related beliefs may help explain gender differences in adolescent depressive symptoms if these beliefs are more strongly linked with depressive symptoms for girls than boys. To test this possibility, gender was examined as a moderator of the association between control-related beliefs and depressive symptoms. 

Three models were tested, moving from a general model to more specific models, in order to understand whether information about the type of life event experienced (i.e., violent or nonviolent) or the type of cognition (i.e., control or contingency) improved prediction of depressive symptoms. The first model, the *General Model*, examined whether adverse life events predicted depressive symptoms and whether control-related beliefs mediated this relationship. The second model, the *Separate Life Events Mode*l, examined violent and nonviolent life events separately to determine whether these types of life events predicted depressive symptoms differently; it was expected that nonviolent life events would be more strongly associated with depressive symptoms than violent life events [14]. The third and most specific model, the *Separate Life Events and Separate Beliefs Model*, examined whether nonviolent and violent life events predicted depressive symptoms and whether control and/or contingency beliefs mediated these associations. Like Model 2, this model examined violent and nonviolent life events separately. In addition, this model examined control and contingency beliefs as separate mechanisms to help clarify the mixed findings for the C-C-C model [18, 20]; specifically, this model tests whether personal control has a stronger association with depressive symptoms than beliefs about group level control (contingency beliefs). By examining specific types of life events (i.e., violent and nonviolent) and mechanisms (i.e., control versus contingency beliefs) that have been deemed important in the development of depressive symptoms, this research informs our understanding of the etiology of depression in African American adolescents.

## 2. Method

### 2.1. Participants and Sampling Design

Participants were drawn from a larger study that evaluated two school-based preventive interventions targeting early learning and aggressive, disruptive behavior [27]. Three first grade classrooms in each of nine Baltimore City public elementary schools were randomly assigned to one of the intervention conditions or to a control condition. The interventions were provided over the first grade year. Of the 678 children who participated in the intervention in the Fall of 1993, 585 (86.3%) were African American. Of the 585 African American children who participated in grade 1, 76% (*N* = 447) completed face-to-face interviews in the 6th grade and 7th grade and reported about their experiences with adverse life events, control-related beliefs, and depressive symptoms. These 447 youth comprised the sample of interest and included 52.6% boys (*n* = 235) and 47.4% girls (*n* = 212). The majority of participants were from lower SES backgrounds, with 72% receiving free or reduced lunch (*n* = 320). At the sixth grade assessment, the mean age of participants was 11.77 (SD = 0.35) with a range of 10.63 to 13.12 years. There were no differences between the 447 participants included in this study and the 138 children from the original sample who did not provide data in 6th and 7th grade in terms of gender, percentage receiving free or reduced lunches, or intervention condition (*Ps* > .05). *t*-tests indicated no differences between the two groups' 1st grade depressive symptoms, anxiety symptoms, or aggressive behavior (*Ps* > .05). Nine participants who participated in the 6th grade assessment did not provide data in grade 7; there were no differences between these 9 participants and the 447 participants included in the present study in terms of participants' gender, receipt of free or reduced lunch, intervention condition, or age.

### 2.2. Procedure in Grades 6 and 7

Permission for participation was obtained through written informed consent by at least one guardian and assent by the participating youth. Each spring, a team of project interviewers conducted standardized interviews with consented youth who provided assent in a private location within the school. Those youth who had dropped out of school or failed to attend were interviewed at a location of their choice. Face-to-face interviews also were conducted with youth within a 90-mile radius of Baltimore. For youth outside this radius, phone interviews were conducted. Participants reported about their experience of adverse life events, control and contingency beliefs, and depressive symptoms. The timing of measurement of these variables was selected in order to best inform a mediation model. Specifically, in 6th grade, participants reported about life events happening in the past year and their current control and contingency beliefs. Depressive symptoms were examined in 7th grade. The time elapsed between 6th and 7th grade measurement points was one year. The study procedures were approved by the Johns Hopkins University Institutional Review Board.

### 2.3. Measures

#### 2.3.1. Demographics

Information regarding participant age, gender, and receipt of free or reduced lunch (as an indicator of socioeconomic status) was collected. Intervention status (i.e., participation in intervention or control condition in first grade) also was recorded.

#### 2.3.2. Adverse Life Events

Experiences of adverse life events were assessed in 6th grade using a modified version of the Life Events Questionnaire Adolescent Version (LEQ-A; adapted from [28]), a self-report checklist of the occurrence of stressful life events within the last year. The LEQ-A was modified for this study in order to include a broader range of life events relevant to adolescence and family-related stressors by adding items from the Adolescent Perceived Events Scale (APES) [29] and the Adolescent-Family Inventory of Life Events and Changes (A-FILE) [30]. In addition, the LEQ-A was modified to allow a test of the “cost of caring” hypothesis; according to this hypothesis, women may be more likely than men to be distressed by another's adversity (e.g., friends, family) in addition to their own, thus leading to higher rates of depression in women [31, 32]. For this reason, the LEQ-A was modified for this study in order to assess three sets of events including: (1) events experienced directly; (2) events experienced by family members; (3) events experienced by friends. Given prior research showing that family and peer life events are salient adolescent stressors that impact youth psychological adjustment, all three types of events (i.e., self, family, and peer events) were included in the study [9, 33]. This life events measure assesses discrete and identifiable life events such as witnessing or being a victim of neighborhood violence, parental job loss, and eviction from the home. The sum of all adverse life events endorsed (“yes” = 1; “no” = 0) was used as an indicator of individual stress levels for Model 1. The sum of nonviolent events and the sum of violent life events were used in the more specific models (i.e., Model 2 and Model 3). A sample item of a nonviolent life event is “Did one of your parents lose his or her job this past year?”; a sample item of a violent life event is “were you or a family member shot or stabbed during this past year?”

#### 2.3.3. Depressive Symptoms

 Depressive symptoms were assessed in 6th and 7th grades using the Baltimore How I Feel (BHIF; [27]), a 45-item self-report measure of depressive and anxious symptoms. Adolescents reported the frequency of these symptoms over the last two weeks on a 4-point scale (0 = Never; 3 = Most times). Item content for the depression subscale was designed based on the diagnostic and statistical manual of mental disorders, third edition, revised [34] criteria or drawn from other existing child self-report measures including the hopelessness scale for children [35], the depression self-Rating scale [36], and the children's depression inventory [37]. The major types of depressive symptoms assessed include symptoms of depressed mood (e.g., “I felt very unhappy”), anhedonia (e.g., “I had a lot of fun”), and depressive cognitions (e.g., “I felt that it was my fault when bad things happened”). The internal consistency alpha for the depression subscale was  .82 in 6th grade and  .83 in 7th grade, indicating that this is a reliable measure. The BHIF depression subscale was significantly associated with a middle school diagnosis of major depressive disorder on the diagnostic interview schedule for children IV (DISC-IV), suggesting it is a valid measure of depressive symptoms [38]. 

For this study, a depressive symptom latent variable was created using three item parcels created from 15 items from the BHIF. Item parcels, or the mean of several items assumed to be conceptually similar, are often preferred over observed variables because they are more likely to meet assumptions of maximum likelihood estimation procedures that are used in SEM, provide more precise estimates of parameters because they reduce the complexity of measurement models, and simplify models by reducing the number of parameters [39]. The three depressive symptom item parcels were used as indicators of the depressive symptoms latent variable.

#### 2.3.4. Perceived Control

 Perceptions of control were assessed in 6th grade using a slightly modified version of the Multidimensional Measure of Children's Perceptions of Control [40]. This modified version includes 24 self-report items assessing beliefs about causes of events, including beliefs about whether events are under one's control or the control of external sources (e.g., “I can get really good grades if I try”; “I cannot stay out of trouble no matter how hard I try”; “If other kids are mean to me, I cannot make them stop.”). Modifications included the addition of a behavioral domain and exclusion of a global domain. Thus, this modified measure assesses beliefs about academic, behavioral, and social domains of perceived control. Youth responded using a 4-point Likert scale (0 = Not at all true; 3 = Very true). Internal consistency alphas for the perceived control measure ranged from  .76 to  .79 for 6th and 7th grades. For this study, the academic, behavioral, and social subscales were used as indicators of the perceived control latent variable.

#### 2.3.5. Perceived Contingency

 Perceived contingency was assessed in 6th grade using the perceived contingency scale for children [41], a 30-item self-report measure that focuses on perceived contingencies for people in general (e.g., “Kids can work hard in school and still get bad grades”; “Kids get yelled at even if they behave”; “Some kids are well-liked and some aren't; it doesn't matter how hard they try.”). Academic, behavioral, and social domains were assessed, and youth responded using a 4-point Likert scale (0 = Not at all true; 3 = Very true). According to Weisz et al. [19] internal consistency alphas for the academic, behavioral, and social contingency subscales were  .69,  .75, and  .74, respectively, and  .86 for the full scale [19]. The academic, behavioral, and social contingency subscales were used as indicators of the perceived contingency latent variable.

## 3. Data Analytic Strategy

### 3.1. Structural Equation Modeling

Structural equation modeling (SEM) using M*plus* 5.1 [42] was used to examine the hypothesized associations between study constructs and maximum likelihood estimates were obtained. Overall model fit was evaluated using multiple indicators including: Chi Square, the Comparative Fit Index (CFI), the Tucker Lewis Index (TLI), and the Root Mean Square Error of Approximation (RMSEA). According to [43], goodness of fit Chi-square ratio values (Chi-square to degrees of freedom ratio) less than 3 indicate good model fit. A relatively good fit between the hypothesized model and the observed data was indicated by CFI and TLI above  .95 and RMSEA less than  .06 [44]. For these analyses, study variables assessed the same year (i.e., adverse life events, perceived control, and perceived contingency) were allowed to correlate in order to account for bias resulting from shared method variance. Intervention status, lunch status, and prior depressive symptoms (6th grade) were controlled in all models by regressing 7th grade depressive symptoms on these variables. 

Mediated effects were tested based on guidelines presented by Holmbeck [45], which are based on guidelines by Baron and Kenny [46]. These analyses included examination of the simple association between the predictor (adverse life events) and the outcome (depressive symptoms), controlling for 6th grade depressive symptoms, to test whether there was a significant association in the hypothesized direction. Paths from the predictor (adverse life events) to the mediator (control-related beliefs) and from the mediator (control-related beliefs) to the outcome (depressive symptoms) also were tested. Each path must be significant in the hypothesized direction for mediation to be present. As described in Baron and Kenny's [46] guidelines, if the previously significant simple association between the predictor and the outcome becomes nonsignificant when the mediator is taken into account, there is support for mediation. To provide an additional test of mediation, the Sobel test [47] was used to test the significance of the indirect effect for each model.

Multiple group analysis was used to test gender differences in the association between control-related beliefs and depressive symptoms. For these analyses, the overall fit of the hypothesized model was tested under two conditions: (a) when the path between the control-related beliefs and depressive symptoms was constrained to be equal for boys and girls (i.e., constrained model) and (b) when there were no constraints on the path between control-related beliefs and depressive symptoms for boys and girls, and the association could vary as a function of gender (i.e., freely estimated model). A significant improvement in model fit, indicated by a significant difference in Chi-square model fit between the free and constrained model, suggests that gender moderates the association between control-related beliefs and depressive symptoms.

## 4. Results

### 4.1. Descriptive Statistics

Means, standard deviations, and ranges for the total sample and separately by gender are presented in Table 1. Boys (*M* = 2.52) reported significantly more total experiences of adverse life events than girls (*M* = 2.07), *t* = 2.25, *P* < .05. Boys (*M* = 1.67) reported more experiences of violent life events than girls (*M* = 1.37); this difference was marginally significant (*t* = 1.95, *P* = .052). Boys (*M* = 7.93) and girls (*M* = 7.94) reported similar levels of experiences of nonviolent life events (*t* = −0.025, *P* > .05). Almost all boys (97.8%) and girls (99.2%) in the sample reported experiencing at least one nonviolent life event. More than half of boys (72.2%) and girls (58.2%) reported experiencing at least one violent life event. Boys (*M* = 43.93) and girls (*M* = 44.29) reported similar levels of control beliefs (*t* = −0.56, *P* > .05). Boys (*M* = 37.46) and girls (*M* = 36.92) also reported similar levels of contingency beliefs (*t* = 0.81, *P* > .05). Reports of 7th grade depressive symptoms were similar for boys (*M* = 10.57) and girls (*M* = 11.54) (*t* = −1.39, *P* > .05). Correlations among all study variables are presented in Table 2. Adverse life events were significantly positively correlated with 7th grade depressive symptoms for boys and girls. While life events were negatively correlated with control and contingency beliefs for girls, they only were significantly related to contingency beliefs for boys. Control and contingency beliefs were positively correlated for boys and girls. Both control and contingency beliefs were negatively correlated with 7th grade depressive symptoms for boys and girls.

#### 4.1.1. Mediation Analyses

Three sets of mediation analyses were conducted: (1) the *General Model*, mediation of the association between adverse life events and 7th grade depressive symptoms by control-related beliefs (control and contingency together); (2) the *Separate Life Events Model*, mediation with violent and nonviolent life events examined separately; (3) the *Separate Life Events and Separate Beliefs Model*, mediation with violent and nonviolent life events examined separately, and control and contingency beliefs examined separately. These analyses were performed using latent variables representing the control-related beliefs and 7th grade depressive symptoms. The *General Model* used a life event latent variable while the *Separate Life Events Model *and *Separate Life Events and Separate Beliefs Model* used two observed variables, nonviolent life events and violent life events. The control-related beliefs latent variable used in the *General Model* and the *Separate Life Events Model* was created from three control beliefs subscales and three contingency beliefs subscales. The perceived control and perceived contingency latent variables used in the *Separate Life Events and Separate Beliefs Model* were created from their respective subscales. Fit indices for the saturated models (all paths included) are summarized in Table 3. Prior to conducting the mediation analyses, measurement models were examined to determine the adequacy of loadings of the indicators on the life events, control-related beliefs, perceived control, perceived contingency, and 7th grade depressive symptoms latent variables to be used in the SEM. All loadings of the indicators for the latent variables in all models were significant (*Ps* < .001).

### 4.2. General Model

**Figure 1:**
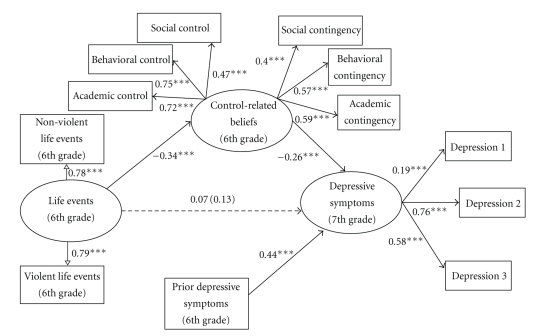
Mediation analyses for the *General Model*. The simple association between 6th grade life events and 7th grade depressive symptoms is in parentheses. This model explained 35% of the variance in 7th grade depressive symptoms (*R* ^2^ = 0.353, *P* < .001). ***P < .001.

The association between life events and 7th grade depressive symptoms, controlling for 6th grade depressive symptoms, lunch status, and intervention status, was tested first and this model was a good fit to the data: (*χ* ^2^(13) = 24.43, *P* < .05; CFI = 0.98; TLI = 0.96; RMSEA = 0.04). There was a marginally significant association between life events and 7th grade depressive symptoms (*β* = 0.13, *P* = .068). Although the association between life events and 7th grade depressive symptoms was only marginally significant, mediation was still possible; therefore, the full model was examined. The full model (Figure 1), with paths from adverse life events to 7th grade depressive symptoms, from adverse life events to control-related beliefs, from control-related beliefs to 7th grade depressive symptoms, and from prior depressive symptoms (6th grade) to 7th grade depressive symptoms (*χ* ^2^(68) = 248.77, *P* < .001; CFI = 0.86; TLI = 0.81; RMSEA = 0.08) explained 35% of the variance in 7th grade depressive symptoms (*R* ^2^ = 0.353, *P* < .001). Intervention status (*β* = −0.01) and lunch status (*β* = 0.09) were not associated with 7th grade depressive symptoms (*Ps* > .05). There was a significant, positive association between 6th grade depressive symptoms and 7th grade depressive symptoms (*β* = 0.44, *P* < .001). The path from adverse life events to control-related beliefs (*β* = −0.34, *P* < .001) and the path from control-related beliefs to 7th grade depressive symptoms (*β* = −0.26, *P* < .001) were significant in the hypothesized direction. The association between adverse life events and 7th grade depressive symptoms was reduced when including control-related beliefs in the model (*β* = 0.07, *P* = .316), suggesting that control-related beliefs mediated the association between adverse life events and 7th grade depressive symptoms. The Sobel test [47] was used to test the adverse life events **→** control-related beliefs **→** 7th grade depressive symptoms indirect path. Results indicated that the adverse life events **→** control-related beliefs **→** 7th grade depressive symptoms indirect effect was significant (*z* = 3.00, *P* < .01).

To test whether gender moderated the association between control-related beliefs and 7th grade depressive symptoms, a model in which this path was freely estimated for boys and girls (*χ* ^2^ = 343.311) was compared to a model in which this path was constrained to be equal for boys and girls (*χ* ^2^ = 344.995). The freely estimated and constrained models were not significantly different (*χ*_diff_^2^(1) = 1.68, *P* > .05), indicating that gender does not moderate the association between control-related beliefs and 7th grade depressive symptoms.

### 4.3. Separate Life Events Model

**Figure 2:**
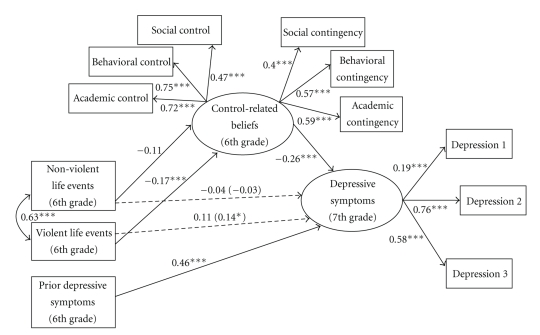
Mediation analyses for the *Separate Life Events Model*. The simple associations between 6th grade nonviolent and violent life events and 7th grade depressive symptoms are in parentheses. This model explained 35% of the variance in 7th grade depressive symptoms (*R* ^2^ = 0.346, *P* < .001). *P < .05; **P < .01; ***P < .001.

The associations between nonviolent and violent life events and 7th grade depressive symptoms, controlling for 6th grade depressive symptoms, lunch status, and intervention status, were examined first. The model fit indices for these simple associations indicated good model fit: *χ* ^2^(10) = 21.63, *P* < .05; CFI = 0.95; TLI = 0.91; RMSEA = 0.05. There was a significant association between violent life events and 7th grade depressive symptoms (*β* = 0.14; *P* < .05), but no significant association between nonviolent life events and 7th grade depressive symptoms. The full model (Figure 2), with paths from nonviolent life events and violent life events to 7th grade depressive symptoms, from nonviolent and violent life events to control-related beliefs, from control-related beliefs to 7th grade depressive symptoms, and from 6th grade depressive symptoms to 7th grade depressive symptoms (*χ* ^2^(64) = 254.80, *P* < .001; CFI = 0.80; TLI = 0.75; RMSEA = 0.08) explained 35% of the variance in 7th grade depressive symptoms (*R* ^2^ = 0.346, *P* < .001). Intervention status (*β* = −0.01) and lunch status (*β* = 0.09) were not significantly associated with 7th grade depressive symptoms (*Ps* > .05). The association between 6th grade depressive symptoms and 7th grade depressive symptoms was significant (*β* = 0.46, *P* < .001). The path from violent life events to control-related beliefs was significant in the hypothesized direction (*β* = −0.17, *P* < .01), but there was no significant association between nonviolent life events and control-related beliefs (*β* = −0.11, *P* > .05). The path from control-related beliefs to 7th grade depressive symptoms was significant in the hypothesized direction (*β* = −0.26, *P* < .001). The association between nonviolent life events and 7th grade depressive symptoms remained nonsignificant. The previously significant simple association between violent life events and 7th grade depressive symptoms became nonsignificant when control-related beliefs were included in the model, suggesting mediation. To provide an additional test for mediation, the indirect path from violent life events **→** control-related beliefs **→** 7th grade depressive symptoms was tested using the Sobel test [47]. The Sobel test [47] indicated that the violent life events **→** control-related beliefs **→** 7th grade depressive symptoms indirect effect was significant (*z* = 2.13, *P* < .05).

To test whether gender moderated the association between control-related beliefs and 7th grade depressive symptoms, a model in which this path was freely estimated for boys and girls (*χ* ^2^ = 340.942) was compared to a model in which this path was constrained to be equal for boys and girls (*χ* ^2^ = 342.227). The freely estimated and constrained models were not significantly different (*χ*_diff_^2^(1) = 1.29, *P* > .05), suggesting that gender does not moderate the association between control-related beliefs and 7th grade depressive symptoms.

### 4.4. Mediation Analyses for the Separate Life Events and Separate Beliefs Model

**Figure 3:**
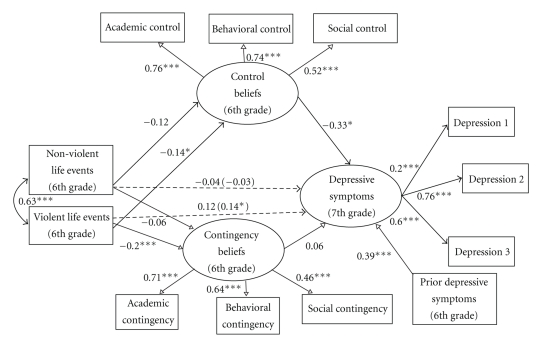
Mediation analyses for the *Separate Life Events and Separate Beliefs Model*. The simple associations between 6th grade nonviolent and violent life events and 7th grade depressive symptoms are in parentheses. This model explained 40% of the variance in 7th grade depressive symptoms (*R* ^2^ = 0.401, *P* < .001). *P < .05; **P < .01; ***P < .001.

The associations between nonviolent and violent life events and 7th grade depressive symptoms, controlling for 6th grade depressive symptoms, lunch status and intervention status, were examined first. The model fit indices for these simple associations indicated good model fit: *χ* ^2^(10) = 21.63, *P* < .05; CFI = 0.95; TLI = 0.91; RMSEA = 0.05. There was a significant simple association between violent life events and 7th grade depressive symptoms (*β* = 0.14, *P* < .05), but no significant simple association between nonviolent life events and 7th grade depressive symptoms (*β* = −0.02, *P* > .05). The full model (Figure 3), with paths from nonviolent life events and violent life events to 7th grade depressive symptoms, from nonviolent and violent life events to perceived control and perceived contingency beliefs, from perceived control and perceived contingency beliefs to 7th grade depressive symptoms, and from 6th grade depressive symptoms to 7th grade depressive symptoms (*χ* ^2^(58) = 139.21, *P* < .01; CFI = 0.92; TLI = 0.88; RMSEA = 0.06) explained 40% of the variance in 7th grade depressive symptoms (*R* ^2^ = 0.401, *P* < .001)*. *Intervention status (*β* = −0.01) and lunch status (*β* = 0.09) were not associated with 7th grade depressive symptoms (*Ps* > .05). There was a significant association between 6th grade depressive symptoms and 7th grade depressive symptoms (*β* = 0.39, *P* < .001). The paths from nonviolent life events to perceived control beliefs (*β* = −0.12, *P* > .05) and from nonviolent life events to perceived contingency beliefs (*β* = −0.06, *P* > .05) were not significant. The association between nonviolent life events and 7th grade depressive symptoms remained nonsignificant (*β* = −0.04, *P* > .05). The paths from violent life events to perceived control beliefs (*β* = −0.14, *P* < .05) and from perceived control beliefs to 7th grade depressive symptoms (*β* = −0.33, *P* < .05) were significant in the hypothesized directions. The path from violent life events to perceived contingency beliefs (*β* = −0.20, *P* < .01) also was significant in the hypothesized direction, but the path from perceived contingency beliefs to 7th grade depressive symptoms was not significant. In this model, the previously significant simple association between violent life events and 7th grade depressive symptoms was not significant. Thus, perceived control beliefs emerged as a possible mediator for the association between violent life events and 7th grade depressive symptoms. To provide an additional test for mediation, the Sobel test [47] was used. The Sobel test [47] indicated that the violent life events **→** perceived control beliefs **→** 7th grade depressive symptoms indirect effect was not significant (*z* = 1.54, *P* > .05).

To test whether gender moderated the associations between perceived control beliefs and 7th grade depressive symptoms, a model in which this path was freely estimated for boys and girls (*χ* ^2^ = 219.560) was compared to a model in which this path was constrained to be equal for boys and girls (*χ* ^2^ = 220.063). The model with the path from perceived control beliefs to 7th grade depressive symptoms constrained to be equal for boys and girls was not significantly different than the freely estimated model (*χ*_diff_^2^(1) = 0.50, *P* > .05). To test whether gender moderated the associations between perceived contingency beliefs and 7th grade depressive symptoms, a model in which this path was freely estimated for boys and girls (*χ* ^2^ = 219.560) was compared to a model in which this path was constrained to be equal for boys and girls (*χ* ^2^ = 219.565). The model in which the path from perceived contingency beliefs to 7th grade depressive symptoms was constrained for boys and girls was not significantly different than the freely estimated model (*χ*_diff_^2^(1) = 0.005, *P* > .05). These results suggest that gender does not moderate the association between perceived control beliefs and 7th grade depressive symptoms or the association between perceived contingency beliefs and 7th grade depressive symptoms.

## 5. Discussion

Research regarding the role of life events in depression development for urban African American adolescents has yielded mixed results [2–5], and little is known about the mechanisms that influence the development and maintenance of depression for urban African American youth [4]. The present study examined whether control-related beliefs mediate the association between adverse life events and depressive symptoms in a sample of urban, low-SES, African American adolescents, and whether gender moderated the association between control-related beliefs and depressive symptoms. Both general and specific relationships between adverse life events, control-related beliefs, and depressive symptoms were examined. 

### 5.1. The General Model

Results from the *General Model* revealed that control-related beliefs mediated the association between adverse life events and depressive symptoms, and a significant adverse life events **→** control-related beliefs **→** depressive symptoms indirect effect. Although these results should be interpreted with caution because there only was a tendency for significance for the original simple association between adverse life events and depressive symptoms, these results suggest that examining general control-related beliefs is a useful way to understand the role of control-related cognitions in the development of depressive symptoms in African American youth. Findings suggest that it may be appropriate for treatment interventions addressing youth depressive symptoms to target both types of cognitions together when working with urban, African American youth. However, in order to provide a clearer understanding of the role of life events and cognitions in youth depressive symptom development, more specific models were examined.

### 5.2. The Separate Life Events Model

Results from the *Separate Life Events Model *indicated that violent life events were significantly associated with depressive symptoms, but nonviolent life events were not. This is counter to some previous research indicating that experiences with violent life events are related to youth externalizing symptoms while nonviolent life events are related to internalizing symptoms in youth (e.g., [14]). These findings indicate that violent life events may be an appropriate screener for urban, African American youth at risk for developing depressive symptoms. Moreover, these findings highlight the importance of examining violent and nonviolent life events separately, in contrast to much prior research that has examined total life events experienced, without examining the differential impact of life events by type. 

Violent life events, rather than nonviolent life events, also may have predicted depressive symptoms because it may be difficult for youth to find effective coping strategies to deal with violent life events as compared to nonviolent life events. For example, for urban youth exposed to violence, strategies such as approach coping may be useful in benign situations but may be less adaptive in violent situations when these coping strategies might lead to negative outcomes, such as future violence exposure and greater distress [48, 49]. In a recent study, Brady et al. [50] found that among African American and Latino boys who had been exposed to community violence, those who had an array of coping strategies, such as modifying behavior without confronting others, had positive long-term adjustment, including less distress, compared to those who had limited coping strategies. For individuals exposed to violent life events then, it may be useful to have an array of coping strategies that they can call upon in order to protect them from negative outcomes. Relatedly, it may be beneficial for depression intervention efforts targeting urban youth to include components that teach youth various coping strategies that they can use in violent as well as nonviolent situations. 

Findings showed that control-related beliefs are one mechanism through which violent life events impact youth depressive symptoms. Urban, African American youth living in dangerous settings, may be particularly vulnerable to feelings that they and others like them have little control over their environments because they may often experience stressors that they may in fact have little control over (e.g., community violence exposure), which in turn impacts their psychological adjustment. In fact, researchers have argued that because inner-city youth are often exposed to pervasive and uncontrollable stressors on a daily basis, they may be especially prone to believe that they have little control over events in their lives [4, 23]. Although this perception may in many instances be realistic, it likely has a negative impact on youth's future responses to adverse life events and can lead to increased depressive symptoms [4]. Results from the present study suggest that urban, African American youth may benefit from learning ways to identify which areas of their lives they in fact do have control over, as well as learning various coping strategies to manage situations in which they have little control (e.g., neighborhood violence).

### 5.3. The Separate Life Events and Separate Beliefs Model

Findings from the *Separate Life Events and Separate Beliefs Model* indicated that examining perceived control and perceived contingency beliefs separately was not a useful way to examine the role of cognitions in youth depression development. Specifically, while there was initial evidence that perceived control beliefs mediated the association between violent life events and depressive symptoms, a test of the indirect effect was not significant, suggesting no mediation. Thus, it seems that examining perceived control and perceived contingency beliefs together is a more useful approach to understanding the development of African American youth's depressive symptoms than examining these beliefs separately.

### 5.4. Which Model Is Most Informative?

In these data, the *Separate Life Events Model* performed better than the *General Model* and *the Separate Life Events and Separate Beliefs Model. *First, by examining life events separately, this model provided useful information regarding the varied utility of different types of life events (nonviolent versus violent) in predicting youth depressive symptoms (compared to the *General Model*). However, the most specific model, the *Separate Life Events and Separate Beliefs Model,* which separated control and contingency beliefs, did not provide additional information regarding the mechanisms involved in depression development. Therefore, the *Separate Life Events Model *is considered to be the superior model in this study.

### 5.5. Gender as a Moderator of the Association between Control-Related Beliefs and Depressive Symptoms

It was hypothesized that the association between control-related beliefs and depressive symptoms would be stronger for girls than boys as previous research has shown that gender differences in cognitive styles, such as increased use of rumination in girls, help to explain the gender difference in depression [24–26]. However, gender did not moderate the association between control-related beliefs and depressive symptoms in this sample. One explanation for this finding is that, unlike rumination, boys and girls may have a similar response to feeling that they have little control over desired outcomes. In other words, low-perceived control over one's environment and negative evaluations of one's abilities, capacities, and worth (i.e., control beliefs) may have similar relations to adolescent psychopathology for both boys and girls [21]. Future research should attempt to further our understanding of how control-related beliefs lead to depressive symptoms in boys and girls.

### 5.6. Implications

Findings suggest that prevention efforts targeting low-income urban youth should assess recent experiences of adverse life events as a means to screen individuals at risk for depression [2]. These interventions should pay special attention to individuals who have experienced violent life events since these types of events seem to play a crucial role in the development of depressive symptoms for urban youth. Findings also suggest that interventions should target control and contingency beliefs. Interventions that focus on individuals' maladaptive cognitions, such as cognitive behavioral therapy (CBT), a well-established treatment for adolescent depression [51], can be used to modify youth's control and contingency cognitions. Depression intervention and prevention efforts also should focus on helping adolescents prepare for and manage experiences of violent life events. Helping youth develop new and effective coping strategies for dealing with experiences with violence may be one way to provide them the tools they need to deal with these frequent stressors. However, more research is needed to understand which coping strategies may be “adaptive” for the contexts in which urban youth live [49]. For example, while studies have generally found that approach coping is linked to better psychological adjustment than avoidant coping, recently there has been increasing recognition that this may not be true for all populations [48, 49]. It may be more adaptive for youth residing in dangerous settings to use avoidant coping than to approach the problem; for example, if a youth approaches a dangerous situation they may be more likely to encounter other negative outcomes such as exposure to violence (Grant et al. [52]). Therefore, future research should examine the utility of different types of coping strategies for urban, African American youth residing in dangerous neighborhoods.

### 5.7. Strengths and Limitations

This study adds to the existing body of research on life events and depression symptom etiology in a number of ways. This study used a prospective, longitudinal design with a sample of predominantly low income, African American adolescents. Due to this design, this study was able to address questions of causality and directionality, more so than previous cross-sectional studies with ethnically diverse youth (e.g., [4]). This study also addressed the potential confounding effect of including cognitive appraisals of life events when assessing stress by using a frequency count of life events without inclusion of cognitive appraisal. This methodology allowed the examination of the individual effects of stressors and cognitions on the development of depressive symptoms and testing of mediational hypotheses. This study also provides a better understanding of the etiology of depressive symptoms in an understudied population, urban, African American youth.

The study strengths should be considered in the context of some limitations. The reliance on self-report measures for assessing experiences of adverse life events, control-related beliefs, and depressive symptoms can be problematic because self-report measures may be vulnerable to response bias. For example, youth's depressive symptoms may affect their reports of experiences of adverse life events. While it would have been advantageous to include multiple informants to assess experiences of adverse life events, it should be noted that other informants may be less beneficial when assessing depressive symptoms and control-related beliefs, since these constructs are not readily observable by others, including parents [18].

### 5.8. Future Directions

These findings suggest several directions for future work. Research has found a positive association between life event stress and youth externalizing behaviors such as aggression, hyperactivity, and conduct problems [53]. Future studies should examine whether control-related beliefs mediate the association between adverse life events and externalizing behaviors in low-income, African American youth. Future research also should examine whether life event stress experienced by youth leads to negative outcomes in general or whether specific types of life event stress lead to specific negative outcomes. There is some evidence that the types of life events experienced by youth are differently associated with internalizing versus externalizing behaviors. For example, in their review of studies examining specificity of stressors on youth psychological outcomes, McMahon et al. [54] found several studies that reported specific relationships between exposure to violence and externalizing outcomes and between divorce or marital conflict and internalizing outcomes. For urban, African American youth, it is important to examine how adverse life events may impact externalizing symptoms in addition to internalizing symptoms. For example, for African American adolescents who reside in neighborhoods characterized by high levels of community violence, it may be more adaptive to display distress through externalizing behaviors than internalizing behaviors [11]. Thus, future research should examine internalizing and externalizing symptoms, including the possibility of co-occurring symptoms. 

Another direction for future research is to examine whether other cognitions influence depressive symptoms. For example, like control-related beliefs [18, 20], cognitions such as self-efficacy beliefs also may play an important role in adolescent depressive symptoms for urban, low-income youth. Researchers should also work to further understand when control-related beliefs begin to play an important role in depression development. Because youth experience cognitive changes such as increased egocentrism and heightened self-consciousness in early adolescence [55], it will be useful to further explore when the effects of control-related beliefs on depressive symptoms begin to emerge, as this information will not only increase our understanding of the etiology of depressive symptoms in youth but also help inform depression prevention and intervention efforts.

## Figures and Tables

**Table 1:** Mean and standard deviations of study variables for total sample and by gender.

	Total		Boys	Girls	*t-*test
Variable	*M* (SD)	Range^a^	*M* (SD)	*M* (SD)	
Life events (6th)	2.30 (2.14)	0–14	2.52 (2.32)	2.07 (1.90)	2.25*
Control (6th)	44.11 (6.92)	18–54	43.93 (6.95)	44.29 (6.89)	−0.56
Contingency (6th)	37.20 (7.03)	13–54	37.46 (6.84)	36.92 (7.23)	0.81
Depressive symptoms (7th)	11.02 (7.66)	0–51	10.57 (6.82)	11.54 (8.50)	−1.39

Note. *N* = 456 for life events grade 6; *N* = 456 for control grade 6; *N* = 456 for contingency grade 6; *N* = 474 for depressive symptoms grade 7.

^a^Refers to observed range.

*P < .05.

**Table 2:** Correlations of study variables for boys and girls.

Variable	1	2	3	4	5	6	7	8
(1) Life events (6th)	—	−.20**	−.13	.42**	.25**	.01	−.05	.14*
(2) Control (6th)	−.19**	—	.49**	−.51**	−.45**	−.01	.08	.06
(3) Contingency (6th)	.21**	.56**	—	−.27**	−.26**	−.11	.04	.02
(4) Depressive Sx (6th)	.24**	−.44**	−.32**	—	.58**	−.01	−.15*	.03
(5) Depressive Sx (7th)	.17**	−.36**	−.31**	.55**	—	−.00	−.10	.16*
(6) Age (6th)	.01	−.11	−.02	−.03	.05	—	−.07	.10
(7) Intervention status	−.10	−.03	−.05	.03	.04	.01	—	.04
(8) Lunch status	.00	−.01	.02	.04	.10	.04	−.22**	—

Note. Correlations for girls are above the diagonal. Correlations for boys are below the diagonal. Sx refers to symptoms. *N* = 447 for life events grade 6, control grade 6, contingency grade 6, depressive symptoms grade 6, depressive symptoms grade 7, age grade 6, and intervention status; *N* = 444 for lunch status.

*P < .05. **P < .01 (1-tailed).

**Table 3:** Goodness-of-fit indices for mediation—saturated models (all paths included).

Model	*χ* ^2^/*df *	CFI	TLI	RMSEA
Saturated general model	3.66	0.86	0.81	0.077
Saturated separate life events mode	3.98	0.80	0.75	0.082
Saturated separate life events and separate beliefs model	2.40	0.92	0.88	0.056

Note. CFI: Comparative Fit Index; TLI: Tucker-Lewis Index; RMSEA: Root mean square error of approximation.
